# Supplementary material for: Are carbon emissions trading and green financial instruments synergistic? -Comprehensive quantitative research based on content analysis
Source: PLoS One. 2024 Mar 7;19(3):e0298601. doi: 10.1371/journal.pone.0298601 (PMC10919601; doi:10.1371/journal.pone.0298601)
Supplement: S1 Data — (ZIP) [file pone.0298601.s001.zip › Data/地级市Z_F工作报告.zip!/╔╜╢1⁄2/═■║ú╩╨/└┤╫╘╣1⁄2╓┌║┼í╛┬φ┐╦╩2╛▌═°í┐_2019.docx]

各位代表：

　　现在，我代表威海市人民政府向大会报告工作，请予审议，并请市政协各位委员提出意见。

一、2018年政府工作回顾

　　过去的一年是极不平凡的一年，习近平总书记亲临威海视察，全市人民倍受鼓舞。在市委的坚强领导下，市政府和全市人民一道，以习近平新时代中国特色社会主义思想为指导，认真贯彻党的十九大和十九届二中、三中全会精神，深入落实习近平总书记视察山东重要讲话、重要指示批示精神，全面落实省委、省政府决策部署，牢牢把握争当全省走在前列排头兵的目标定位，按照“1+33333”工作体系，加快建设“精致城市·幸福威海”。

　　（一）强化“一个统领”，加快推动新旧动能转换。坚持把新旧动能转换作为统领经济工作的重大工程，把推进供给侧结构性改革与“六稳”工作有机结合，推动高质量发展取得新进展。

　　发展质量效益有新提高。经济运行稳中向好、结构趋优。全市生产总值3641.5亿元，增长6.7%，三次产业比重由7.7:45:47.3调整为7.7：44.0:48.3，高新技术产业产值比重由41%提高到45.1%。一般公共预算收入284.4亿元，增长4.2%，剔除不可比因素，增长9%；税收占比达到81%，提高1.7个百分点。新增城镇就业4.1万人，城镇登记失业率1.81%，保持全省较低水平；金融机构贷款余额增长13.5%，不良贷款率保持全省较低水平；实际到账外资8.6亿美元，增长5.5%；固定资产投资增长7.5%，“六稳”工作扎实有效。

　　产业集群化发展有新突破。建立“五个一”协调推进体系，加快建设十大高端特色产业园区，集中力量培育七大千亿级产业集群，5个产业集群入选全省首批现代优势产业集群，高区被认定为医疗器械国家新型工业化产业示范基地。实施139项新旧动能转换重大项目、187项市级重点项目、147项市级重点工业投资项目、133项重点科技创新项目，惠普3D打印中心项目落地，亿和精密加工、捷普电子、正崴打印设备、康得碳谷科技、马夸特电动开关、南海碳材等重点项目加快推进。获批创建省级中医药健康旅游示范区，旅游消费总额增长12.4%。

　　新经济培育有新亮点。实施智能制造专项行动计划，迪尚集团列入国家智能制造专项、高级定制体验中心投入运营，天润曲轴入选全省智能制造标杆企业，新增智能工厂和数字化车间7家，“上云”企业达到560家。捷讯通信、成山轮胎入选全省服务型制造“1+N”示范企业。2件作品获得“省长杯”工业设计大赛金奖。金猴集团、威高血液、泓淋电力等3家企业入选国家级制造业单项冠军培育企业，浩然特塑、盈盾科技等7家企业入选首批全省瞪羚企业示范培育名单，腾森轮胎、浦源食品等10家企业入选全省中小企业隐形冠军企业，总数达到34家。

　　海洋经济发展有新起色。强化“五个导向”，推进海洋强市建设，获批国家海洋经济发展示范区，新增2处省级科技兴海产业示范基地。国家海洋经济创新发展示范城市建设加快推进，实施产业链协同创新项目24个。新增省级海洋牧场示范项目9个，休闲渔业产值增长15.1%。压缩近海捕捞产能1.8万千瓦，远洋捕捞产量占捕捞总产量的比重超过40%。

　　创新发展活力有新增强。按照做实“1”、做强“3”、做活“N”的思路，推动国家区域创新中心公司化、实体化，工信部电子信息技术综合研究中心引进12个高端平台项目，哈工大威海创新创业园新增2个研究院，高性能医疗器械创新中心建立6个技术创新平台。光威复材获批国家企业技术中心，新增省级企业研发平台32家。举办首届全国医疗器械科技创新大会、科技创新暨成果转化合作大会等产学研对接活动，新增产学研合作战略联盟4个。新引进技术转移中心10个，中澳先进制造业联合研究中心建成启用。成功举办第十届“海洽会”暨威海“英创会”，深化提升威海英才计划，新增国家千人计划等领军人才54名、各类高技能人才2083人。获得国家科技进步二等奖3项。全社会研发投入占生产总值比重由2.53%提高到2.66%。

　　“三城联创联建”有新成效。推动国家质量强市示范城市、国家知识产权示范城市、国家社会信用体系建设示范城市“三城联创联建”，质量强市建设的典型做法被国务院通报表扬。成立全国碳纤维产业计量测试联盟，新增省级以上检验检测中心3家。威高集团获批建设国家医疗器械技术标准创新基地，威力工具通过全省首批“泰山品质”认证，三角轮胎获评国家质量标杆企业、国家知识产权示范企业、国家技术创新示范企业，12家企业获评国家知识产权优势企业。加强社会信用体系建设，开展“海贝分”个人信用积分，在299个行政管理事项中推行联合奖惩。

　　（二）实施“三大战略”，大力推进精致城市建设。习近平总书记提出“威海要向精致城市方向发展”，为我们指明了前进方向，提供了根本遵循。我们以此为引领，大力推进城市国际化，着力提升城市品质内涵，推动城乡区域协调发展。

　　对外开放深度拓展。实施城市国际化战略行动计划，成功举办首届市长国际经济咨询委员会年会、儒商大会招商推介会、中韩服务贸易高端论坛、首届中韩创新大赛、第七届世界海洋大会、中国欧盟膜技术创新合作大会等重要活动，开展香港周、欧洲周、日韩周等重大经贸活动。设立招商“服务大使”，狠抓招商引资，新增外资项目235个，增长23%。举办国际渔具博览会、国际食品博览会、中韩文博会等重要展会22个，新增国家级外贸转型升级基地4处。深化服务贸易创新发展试点，服务贸易出口增长34%。获批国家级跨境电子商务综合试验区，跨境电商零售出口增长56.6%。深化中韩自贸区地方经济合作示范区建设，对韩进出口增长6.9%，实际到账韩资增长10.9%。设立中德协同创新中心，加入中德工业城市联盟。综合保税区通过封关验收。

　　乡村振兴步伐加快。聚焦“五个振兴”，制定乡村振兴战略规划和三年行动计划。完成520个村的集体产权制度改革，新增土地流转面积5.2万亩，新建农业社会化综合服务中心33处，新增农民专业合作社207家、家庭农场368家，麓原果香田园综合体成为省级田园综合体试点。推进粮食绿色高产高效创建工作，新增省级生态循环农业示范基地6处。完成18个美丽乡村精品示范村建设。荣成市及6个镇、60个村入选全省乡村振兴“十百千”示范县镇村。深入实施涵育文明乡风行动，荣成市入选全国新时代文明实践中心建设试点县。

　　城市环境全面提升。开展城市设计，编制城市家具设施、户外广告等专项设计，提高城市设计管理精细化、标准化水平。扎实推进“城市双修”试点，启动城市短板三年整治行动，打通市区7条瓶颈路，治理背街小巷20处，新建改造城市公厕25处。开工清洁供暖项目437.8万平方米，完成城市海绵功能改造9.6平方公里。开展环境容貌专项治理，治理违法建设430.7万平方米。开展“寻找威海记忆”主题行动，挖掘整理威海“老字号、老技艺、老滋味”，进一步擦亮城市文化名片。

　　区域统筹协调发展。出台政策推动县域创新驱动发展，荣成市入选国家首批创新型县市、全国守信激励创新试点市。环翠区获批省级双创区域示范基地，整合资源打造威海创新园，威海国际人力资源服务产业园获评省级产业园。推动开发区转型升级创新发展，高区获批全国中小企业创新创业升级特色载体，临港区获批中欧（威海）中小企业合作区，经区入选全省现代服务业集聚示范区。深入实施“四个突破”战略，东部滨海新城、南海新区开发和文登同城化、乳山产业结构调整步伐加快。张村镇、宋村镇等7个镇入选全国综合实力千强镇。

　　（三）突出“三个力保”，着力提升城市承载能力。围绕完善规划、健全制度、夯实基础，全力保证交通快速通达，全力保障水资源安全，全力保护海岸线等生态资源。

　　强化规划控制守红线。科学划定“三区三线”，完成城市风貌保护规划编制，搭建三维实景城市模型平台，建设“多规合一”综合运行系统。开展人口综合承载力研究。编制综合交通网中长期发展规划、城市轨道交通建设规划，修订威海湾港区、南海港区总体规划，制定深化港口发展实施方案。编制水安全保障总体规划、湿地保护总体规划、矿山地质环境保护与治理规划，进一步优化生态保护红线。

　　强化建章立制严监管。严格落实河长制，全面建立湖长制，在27个海湾推行湾长制，建成“智慧河长”平台，实现河湖一体化管理。开展“大棚房”集中整治行动。出台生态环境损害赔偿制度改革方案、环境保护约谈办法。制定实施河库绿化、河道治理、山体修复、海岸带修复等三年行动计划，摸清底数、明确目标、细化方案，有序推进生态修复，厚植生态底色。

　　强化工程建设强基础。文莱高速公路等33项交通重点项目加快推进，改造农村公路391公里，荣成市被评为全国“四好农村路”示范县，文登区、乳山市被评为全省示范县。乳山河地下水库工程竣工，龙角山水库、坤龙邢水库增容工程快速推进，完成22座小型水库、206座病险塘坝除险加固工程。实施38个海岸带修复项目，深入推进“六林工程”，治理穿村过镇河道及中小河流150公里，完成河库造林绿化和生态治理2.7万亩。

　　（四）打好“三大攻坚战”，牢牢守住发展的底线红线。强化责任担当，防范化解重大风险、精准脱贫、污染防治三大攻坚战取得阶段性成果。

　　重大风险防范扎实有效。开展金融风险专项排查行动，积极化解重点风险企业、非法集资、互联网金融等领域风险，金融生态环境持续优化。推动化工企业进区入园，完成238家化工企业评级评价工作。加强海上安全管理，清理取缔海洋涉渔“三无”船舶2523艘。建立企业生产经营全过程安全责任追溯制度，安全生产形势保持稳定。开展信访积案化解和排查安全隐患防范“五类风险”专项行动，圆满完成上合组织青岛峰会等重大安保维稳任务。

　　精准脱贫攻坚拓面提标。以“老病残”和“插花式”贫困人口帮扶为重点，在巩固提升180个省定扶贫工作重点村的同时，加强对504个经济薄弱村的帮扶，29.8万群众受益。整合提升扶贫产业项目277个，发放“富民农户贷”“富民生产贷”7.5亿元。严格落实“三保障”政策，为扶贫户购买扶贫特惠保险，帮助4749户贫困群众改善住房条件，将贫困人口全部纳入重特大疾病住院救助和门诊慢性病救助范围，补贴和减免医疗费用。

　　环境污染防治标本兼治。健全长效机制，集中力量整改中央环保督察、国家海洋督察、省级环保督察反馈问题。开展“四减四增”三年行动，主要污染物排放量完成削减任务，万元生产总值能耗降低2.9%。实施“散乱污”企业治理等专项行动，环境空气质量连续三年稳定达到国家二级标准。科学调整12处城市集中式饮用水水源地保护区，编制近岸海域污染防治方案。开展土壤污染状况详查，工业固体废物处置中心开工建设。

　　（五）坚持“三个聚焦”，打好支持实体经济发展组合拳。坚持聚焦区域、聚焦产业、聚焦政策，实施96项省级以上改革试点，优化配置政策“工具箱”，助力民营企业健康发展。

　　助力企业降低成本。全面落实支持民营经济发展系列政策，兑现各项减税降费政策，调整城镇土地使用税适用税额标准，新增直供电试点企业292家，落实阶段性降低保险费率和困难企业缓缴延缴政策，实施失业保险援企稳岗护航行动，通过减收增支为实体经济输血155.6亿元。

　　助力企业融资发展。完善科技支行、风险补偿、融资担保等政策，设立企业应急转贷基金，开展专利权“政银保”融资试点，帮助企业解决融资难题。设立新旧动能转换产业发展基金及4支子基金，总规模300亿元。非金融企业发行债券23支，总额171.3亿元。威高股份获批H股第一批全流通试点，浦林成山在香港上市，新增新三板挂牌企业4家、区域股权市场挂牌企业61家。

　　助力大众创新创业。实施科技企业孵化器聚集提升工程，新增省级科技企业孵化器和众创空间14家，建成工匠人才创新工作室382家。荣成市、文登区被评为省级创业型城市。实施优秀企业家培育计划，举办民营企业家“建功立业新时代”专题研修班。市场主体突破30万户，增长12.9%。新增国家认定的科技型中小企业398家，高新技术企业由297家增加到395家。

　　（六）突出“三个整合”，全面优化营商环境。深化“放管服”改革，加快整合政务服务热线、整合共享政务信息系统、整合政务督查力量，扎实开展营商环境大提升行动。

　　整合政务资源促进高效联动。整合45条政务服务热线，建立全市统一的12345政务服务平台，健全“一号受理、限期办结、综合督办、双向反馈、回访全覆盖”机制，按期办结率、群众满意率分别达到99.5%和91.7%。建成信息资源共享交换平台，市直部门数据归集率达到85.4%，78个部门开展数据交换，40个部门实现数据实时对接。建立经济运行大数据中心，开展大数据创新应用，政务信息系统整合共享经验全省推广。积极整合政务督查力量，创新现场检查、暗访抽查、专家评估等方式，进一步强化执行力，推动各项重点工作落地落实。

　　深化“一次办好”改革利企便民。全面推进审批服务便民化与政务服务“一张网”建设，政务服务“一次办好”事项由739项增加到2079项，占比达到97.3%。开展非公有制经济走访调研活动、群众办事百项堵点疏解行动，集中力量解决企业和群众办事中的难点痛点堵点问题。开展贴心帮办代办省级试点工作，设立投资项目代办服务中心，在全市政务服务大厅全面推行咨询导办、贴心帮办、全程代办服务。市县两级组建行政审批服务局，实现“一枚印章管审批”。率先开展政务服务事项“三同一”工作，精简办理环节972个。开展“证照分离”改革试点。持续推进“简证便民”，取消证明事项30项。开展优化营商环境15个专项行动，企业开办、不动产登记、建设工程项目审批、企业水电气暖报装等流程进一步简化，服务进一步提速。

　　主动转变作风推进依法行政。深入开展“大学习、大调研、大改进”，认真整改中央巡视反馈问题，省委巡视发现问题即知即改。政府机构改革扎实推进，职能配置进一步优化。认真做好政府立法工作，配合市人大常委会开展地方立法，《威海市海岸带保护条例》《威海市文明行为促进条例》等地方性法规正式颁布。自觉接受人大及其常委会法律监督和工作监督，主动接受政协、民主党派民主监督，办理人大代表建议85件、政协提案283件。深入推进政务公开，主动接受社会监督和舆论监督。推行“4+2”意识形态工作责任制，守牢意识形态阵地。加强廉政警示教育和岗位廉政风险排查，进一步改进党风政风。

　　各位代表！习近平总书记到山东考察时强调，民之所盼，政之所向，增进民生福祉是发展的根本目的。我们认真践行以人民为中心的发展思想，扭住人民群众最关心的问题，扎扎实实做好民生工作，努力使人民群众的获得感、幸福感、安全感更加充实、更有保障、更可持续。

　　——不遗余力增强群众的获得感。坚持从“小切口”改善“大民生”，全力办好群众的烦心事、操心事。通过政务服务热线等平台，帮助群众解决身边的难题33万件。以对群众负责的态度，实施城市建设领域矛盾纠纷化解行动，解决城市“成长中的烦恼”，初步摸排出189个历史遗留问题，已解决31个，为1.1万户居民解决了不动产权证办理等实际问题。完成老旧小区整治13个，惠及9769户居民。贴近群众需求办理12件民生实事，建设小游园30处，开工建设城市绿道48.8公里。开通和优化公交线路30条，受益居民小区132个。利用老建筑建设城市博物馆等公益文化空间9处，市群众艺术馆、图书馆新馆建成开放，新建城市书房16处、共享书柜25个。新建全民健身中心9处，实现镇街全覆盖。新增社区智慧便利店68家。新建农村社区服务中心154处，完成农村改厕10.5万户，改造农村公共场所供暖设施875处，新增农村清洁供暖用户9579户，改善提升252个村的居民饮水条件，从点滴小事做起改善农村群众生产生活条件。

　　——不遗余力增强群众的幸福感。致力于满足人民美好生活需要，织密扎牢社会保障网，在幼有所育、学有所教、劳有所得、病有所医、老有所养、住有所居、弱有所扶上不断取得新进展。新建改扩建普惠性幼儿园23所，普惠性幼儿园覆盖率由84.5%提高到87%。新建改扩建中小学校26所，增加学位9750个。实施就业创业培训3.1万人次，城镇和农村居民人均可支配收入分别增长7.5%、7.7%。实施职工长期护理保险制度，提高居民医保财政补助标准，居民大病保险最高补偿限额由30万元提高到40万元。市中心医院病房楼、中医院康复楼投入使用，组建县域医共体4个，城乡居民家庭医生签约服务率分别达到31.4%和65.7%。新建医养结合机构10处，新增居家养老服务组织72个，设立农村区域性养老服务中心13处。制定家政行业团体标准3项，培训家政从业人员7000人次。改造棚户区2.9万套，为447户家庭发放住房租赁补贴。实施残疾人精准康复行动和残疾人家庭无障碍智能化改造，开展慈善大病救助、“夕阳扶老”等救助活动，惠及困难群众7.1万人次。困难群众医疗救助实现“一站式”结算，提高城乡最低保障标准和特困人员补助标准，保障困难群众2.7万人，民生保障标准更高、覆盖面更广。

　　——不遗余力增强群众的安全感。坚持共建共治共享，从基层基础建设抓起，调动群众积极性，共同建设平安威海。健全立体化社会治安防控体系，新增监控探头1.2万台，总量达到27万台。扫黑除恶专项斗争取得阶段性成果，侦办黑社会性质组织案件4起、恶势力犯罪集团案件20起、恶势力犯罪团伙案件28起，刑事案件、治安案件分别下降2.5%和16.4%。深入推进普法和依法治理，拓展法律援助服务网络，调处矛盾纠纷1.6万件。整合肉菜、海参、西洋参、老字号追溯体系，完善威海重要产品追溯管理平台。实施餐饮质量安全提升工程，推行“互联网+明厨亮灶”，建成网络订餐监管平台，守护群众舌尖上的安全。获批创建国家公共文化服务体系示范区，实现文化强省建设先进市、县“全域一片红”；大力弘扬殷树山等“齐鲁时代楷模”精神，推动文明城市建设由“城”的文明向“人”的文明聚焦，营造起文明和谐、平安有序的社会氛围。

　　各位代表！习近平总书记的亲切关怀，温暖着威海人民，激励着我们奋发向上、砥砺前行。全市上下大力弘扬改革开放40年所形成的威海精神，强化创先争优、勇争一流的意识，主动寻标对标，自我加压，高点定位再出发，兴起新一轮解放思想、深化改革、扩大开放热潮；强化雷厉风行、专业务实的作风，大力弘扬新时代泰山“挑山工”精神，凝心聚力干事创业；社会各界同心协力、群策群力，汇聚起攻坚破难、追赶超越的强大合力。在此，我代表市政府，向全市各族人民，向人大代表和政协委员，向各民主党派、工商联和无党派人士、各人民团体，向驻威人民解放军指战员、武警官兵，向各位老领导、老同志，向关心支持威海发展的各界人士和海内外朋友，致以崇高敬意和衷心感谢！

　　我们也清醒地看到，全市经济社会发展还存在一些矛盾和问题，政府工作还存在不少差距和不足。外部环境复杂严峻，经济面临下行压力；新产业、新业态发育不足，新旧动能转换“空笼期”问题不容忽视；大项目、大企业不多，产业集群化、园区化发展水平有待提高；创新驱动能力不强，科技和人才短板依然突出；城市建设不够精致、管理不够精细，影响群众生活的噪声、油烟等环境问题依然不少；民生领域还有不少短板，公共服务供给存在不平衡不充分的问题；风险防控压力加大，社会治理能力需要增强；营商环境仍有很大提升空间，政府系统廉政建设有待进一步加强。我们一定强化忧患意识、责任意识、进取意识，坚持目标导向、问题导向、结果导向，不断加强和改进工作。

二、2019年政府工作的目标和重点

　　各位代表！习近平总书记的殷切嘱托、深切期望，重若千钧，激人奋进。我们要树立责任意识，强化担当精神，以百倍的激情、充足的干劲，把总书记的浓浓关怀、谆谆勉励转化为全市上下干事创业的强大动力，紧紧抓住和用好重要战略机遇期，坚持务实再务实、落实再落实、抓紧再抓紧，推动各项工作取得新突破、再上新台阶。

　　今年是新中国成立70周年，是决胜全面建成小康社会第一个百年奋斗目标的关键之年。按照中央和全省、全市经济工作会议精神，政府工作的总体要求是：以习近平新时代中国特色社会主义思想为指导，认真贯彻党的十九大和十九届二中、三中全会精神，深入落实习近平总书记视察山东重要讲话、重要指示批示精神，把“威海要向精致城市方向发展”作为总目标总方向总遵循，牢牢把握争当全省走在前列排头兵的目标定位，坚持稳中求进工作总基调，坚持新发展理念，坚持推动高质量发展，坚持以供给侧结构性改革为主线，坚持深化市场化改革、扩大高水平开放，以新旧动能转换重大工程为引领，大力实施海洋强市、乡村振兴和城市国际化战略，强化创新驱动，着力培育现代优势产业集群，继续打好三大攻坚战，统筹推进稳增长、促改革、调结构、惠民生、防风险工作，保持经济运行在合理区间，进一步稳就业、稳金融、稳外贸、稳外资、稳投资、稳预期，保持经济持续健康发展和社会大局稳定，全力开创“精致城市•幸福威海”新局面。

　　主要预期目标为：生产总值增长6.5%；一般公共预算收入增长5%，收入质量进一步提升；投资结构持续优化，消费对经济增长的贡献率进一步提高，货物和服务贸易稳中提质;实际到账外资增长5%；城镇和农村居民人均可支配收入增长7%；城镇登记失业率控制在2.5%以内；居民消费价格涨幅3%以内；全面完成省下达的年度节能减排降碳约束性目标和环境质量改善目标。

　　按照“抓住重点、破解难点、补齐短板、树起亮点”的要求，强化“八个聚焦”，冲击新目标，培育新优势，实现新突破：

　　（一）聚焦新旧动能转换，加快推动高质量发展。坚持以供给侧结构性改革为主线不动摇，着力在“巩固、增强、提升、畅通”八个字上下功夫，强化产业为王、项目为王、企业为王，按照项目化、工程化思路，推动新旧动能转换重大工程落实落地。

　　全力扶优培强，激发民营企业发展活力。实施企业冲击新目标行动，助推企业做强主业、做实基础、做大规模，培育更多的主营业务收入过千亿、过百亿、过十亿、过亿元企业，强化正向激励，发展更多优质企业。实施制造业单项冠军企业培育行动，培育15家瞪羚企业和准独角兽企业、50家行业隐形冠军企业，新增60家高新技术企业，支持企业走“专精特新”发展道路。建立企业问题快速会商决策机制，及时协调解决企业经营和项目落地建设中遇到的难题。成立政府投资引导基金管理公司，设立七大千亿级产业集群基金、天使基金和直投基金，发挥杠杆效应，激活民间投资。整合设立市信用保证基金，建立“信财银保”四方联动和风险共担机制，联合为企业融资提供增信服务。扎实开展优化营商环境15个专项行动，全面落实减税降费政策，降低企业用能、用地、物流等各类营商成本，压缩水电气暖报装时间，增加优质服务的可获得性。综合运用企业纾困基金等多种手段，帮助民营企业纾困解难。开展营商环境第三方评价，下大力气帮助企业解决发展中的困难、前进中的问题、成长中的烦恼。

　　强化项目支撑，培育壮大千亿级产业集群。完善“五个一”协调推进体系，实施139项新旧动能转换重大项目、179项市级重点项目，强化龙头企业和示范项目带动，利用技术创新和规模效应形成新的竞争优势。加强细分产业研究，推动七大千亿级产业集群加快向产业链高端迈进、向创新链高端转型、向价值链高端延伸。开展新一轮高水平技术改造三年行动计划，加快企业“零土地”技术改造项目审批方式改革，推动制造业技术改造和设备更新，实施136项市级重点工业投资项目，年内完成投资223亿元。巩固“三去一降一补”成果，加快出清“僵尸企业”，盘活闲置厂房和土地资源，推动“腾笼换鸟”。

　　突出特色优势，加快海洋产业转型升级。落实海洋强省建设十大行动，坚持“五个导向”，实施“5+2+2”海洋产业提升工程，重点推进37个海洋经济创新发展示范项目和143个产业项目建设，壮大海洋发展新动能，建设创新型国际海洋强市。推动威海港与青岛港合作发展，加快两港一体化运营。大力发展临港经济，推动以港兴市、兴港强市。实施18个海洋牧场项目，建设沙窝岛国家级远洋渔业基地等5个远洋渔业项目。加快发展海洋产业基金、海上保险、航运融资和大宗海产品交易，推动海洋金融创新发展。建设威海国际海洋高新技术交易中心，支持省级科技兴海产业示范基地和海洋工程协同创新中心建设，打造智慧海洋大数据中心，促进创新链、产业链深度融合。

　　推动模式创新，培育壮大“四新”经济。推动先进制造业和现代服务业深度融合，开展“制造+服务”外贸转型升级试点，促进新技术、新组织形式、新产业集群形成和发展。大力发展数字经济，深入实施工业互联网战略，实施智能制造引航工程，推进工业互联网云平台建设，建立智能制造产业联盟，完善“机器换人”项目库，推进实体经济与人工智能、云计算、大数据深度融合，“上云”企业数量超过600家。壮大总部经济和楼宇经济，改造提升3处双创特色街区和4处特色商业街区。聚焦满足最终需求，推动家政服务、教育培训、文化娱乐、体育健身等生活性服务业向精细化和高品质转变，进一步改善消费环境。

　　强化全域统筹，提升发展精品旅游。加快创建国家全域旅游示范区，依托幸福海岸、山地休闲两条旅游带，构建滨海观光、休闲度假、温泉养生等相互支撑的多元业态结构。新建综合性游客集散中心，打造展示威海名优产品和旅游特色产品的“一站式”旅游购物场所。引进和推动国际高端酒店建设，规划论证国际邮轮港口，促进海上旅游、会展与钓具渔具、游艇制造等产业联动发展，开发冬季旅游、夜间旅游、工业旅游、康养旅游等系列产品，进一步打响“四季威海·康养福地”品牌。

　　坚持质效优先，实施质量、品牌和标准化战略。继续推进“三城联创联建”，创建全国质量强市示范城市，开展质量提升行动，积极争创钓具、碳纤维及复合材料、纺织服装与时尚创意、智能制造装备等国家产品质量提升示范区。深入开展国家标准化综合改革试点，加快国家医疗器械技术标准创新基地建设，实施145个标准化项目，以高标准引领高质量发展。实施品牌高端化工程，建设“优品威海”品牌宣传推介公共服务平台。启动威海产业创新展示中心建设，打造全市产业发展和科技创新成果展示窗口。深入开展放心消费创建工作，打造“放心消费·诚信威海”品牌。

　　强化协同联动，增强金融服务实体经济能力。强化金融与财政、科技、产业等方面政策协同，推动政府、金融机构与企业协调联动，优化完善科技支行、小微企业贷款风险补偿基金、小微企业贷款保证保险等政策，帮助民营企业和小微企业解决融资难、融资贵问题。推动银行机构建立无还本续贷企业名单，综合利用企业应急转贷资金，帮助企业无缝续贷。优化信贷结构，提高存贷比，推动更多金融资源向民营经济、小微企业、三农聚集。推动企业利用好资本市场，提高直接融资规模和比重，争取新增上市挂牌企业15家。推动金融产业聚集发展，积极引进、设立金融机构和新型金融组织。深化地方金融改革创新，推动地方法人银行机构完善公司治理，鼓励发展政府性融资担保机构，争创国家绿色金融改革试验区。

　　（二）聚焦精致城市建设，做实做细城市特色品牌。注重精当规划、精美设计、精心建设、精细管理、精准服务，实施“精致城市·幸福威海”三年行动。

　　坚持精当规划。强化全域威海观，精当科学统筹空间、规模、产业三大结构，优化城市发展布局。加快推进“多规合一”，启动新一轮国土空间规划编制工作，建立城市规划数据库，形成城乡统筹、全域覆盖、要素叠加的“一张蓝图”，保持“大山海、小珠链”城市风貌空间格局。运用三维实景城市模型平台，推进市域卫星影像及数据更新，对城市建设、管理和运营实施精准管控。划定城市功能分区，明确城市组团、片区、街道的功能定位、风貌风格和体量规模，做好威海老港区、东山宾馆以东疗养区等重点片区的规划设计，推动城市有机更新。建立精致城市评价指标体系。实施城市规划馆优化提升工程，打造城市展示中心。推动文登同城化发展，促进各城市组团协调发展。

　　坚持精美设计。全面开展城市设计，突出生态底色、文化特色和现代元素，精美塑造城市特色风貌。健全城市设计导则，加强重要节点、景观走廊和开敞空间设计，统筹建筑布局，协调城市景观，提高单体建筑设计水平，营造精巧别致的城市空间，让城市显山露水、融入大自然。挖掘威海卫古城文化底蕴，实施历史街区保护开发，保护修缮利用老洋房，延续历史文脉，留住城市记忆，加快创建国家历史文化名城。建设一战华工纪念馆，打造城市国际化展示交流平台。办好第十届中国威海国际人居节及国际建筑设计大奖赛。

　　坚持精心建设。精心组织实施3大类、12大项城建重点工程，着力提升城市功能品质。深入推进“城市双修”，开展扮绿城市、点亮夜景、疏解交通、修复疤痕和开发地下五大专项行动，积极创建国家生态园林城市。统筹绿化、花化、彩化，推进憩园修绿、空地植绿、河道护绿、见缝插绿和拆违补绿，加快主干路绿化景观升级，改造提升威海湾沿线公园的老旧设施，新建小游园32处，推进5处山体公园建设。实施威海湾—刘公岛亮化工程，优化威海湾沿线公园、商业建筑亮化设计，与现有设施环境提升相结合，打造“十里夜威海”美丽时尚海岸线。加快昆明路西延、统一路南延、疏港路立交等城市路网项目建设，推动沿海岸线人行步道贯通，改善城市“微循环”。实施10个老旧小区改造工程，开展公园路等10条背街小巷治理，新建改造城市公厕16座，规范设置管理垃圾箱等城市家具，开展户外广告、门头牌匾专项整治，精准治理“城市伤疤”。统筹开发利用城市地下空间，完善地下管网体系，实施6项海绵城市改造工程，开工建设地下管廊6公里。完成文登生活垃圾焚烧处理项目，启动市垃圾处理厂二期项目建设，2020年实现全域原生生活垃圾“零填埋”。

　　坚持精细管理。强化“城市管理要像绣花一样精细”的理念，从细微处入手，以“小切口”促进城市运行效能大提升。加强智慧城市建设，加快人工智能、大数据、云计算、物联网等新技术在城市管理中的深度运用。开展物业品牌提升年活动，推行物业信用管理。深入开展城市建设领域矛盾纠纷化解行动，实行动态清单管理，集中力量解决城中村改造、半拉子工程、不动产权证办理等困扰群众的历史遗留问题。抓好已经在城镇就业的农业转移人口的落户工作，户籍人口城镇化率提高1个百分点。

　　坚持精准服务。从群众反映的痛点、难点、堵点问题抓起，着力治理“城市病”，打造有温度的精致城市。建成羊亭公交中心站，开通和优化7条公交线路，惠及92个居民小区。支持建设立体停车场，推进市立医院停车场建设，挖潜停车资源，中心城区新增停车位1300个；加强车辆静态管理，缓解城市拥堵。着力打造精致社区，优化升级社区便民服务设施，新建改造社区菜市场4处。市游泳馆建成投入使用，建设威海市民中心、临港区市民中心、南海新区市民中心、环翠区公共文化服务中心，新增面向社会开放体育设施的学校30所，打造城市社区10分钟健身圈，方便群众休闲健身。

　　（三）聚焦城市国际化战略，打造对外开放新高地。牢牢抓住城市国际化战略、双招双引、开放型经济新体制“三个着力点”，深化市场化改革，扩大高水平开放。

　　深化重点区域合作。坚持深耕日韩、拓展欧美、强化“一带一路”、提升港澳台，扩大与京津冀、长三角和珠三角地区合作。按照“三互四港五联通”的思路，与韩国仁川共建协同发展新格局，打造中韩自贸区地方经济合作示范区升级版。推进国家级跨境电子商务综合试验区建设，探索与韩国仁川、日本福冈共同打造区域性跨境电子商务创新试验园区和国际合作服务平台。加强威海—仁川—福冈口岸协作，开展跨境物流多式联运，打造海上合作战略支点。加强与日韩商会协会、大企强企、孵化机构对接，建设韩国科技产业园，设立韩国技术创新研究院，提升中韩创新大赛层次，吸引更多日韩高端项目。优化中欧班列运行，推进威海国际物流多式联运服务中心建设。加快建设中欧（威海）中小企业合作区、中欧水处理及膜技术创新产业园，对接更多“德国工业4.0”项目，争取实现对欧合作新突破。

　　推动高质量双招双引。坚持把双招双引作为经济工作的生命线，各级领导干部带头聚力抓招商，聚焦世界500强、央企百强企业及行业领军企业，聚焦产业发展导向，策划开展专题招商活动30次以上，推动建链、强链、补链项目尽快落地，提高招商引资实效性和精准度。完善考核机制，发挥好国家级、省级开发区和各类园区的招商引资主阵地作用。整合社会化招商资源，培育专业化招商团队，开展产业链招商、委托招商、以商招商、平台招商、乡情亲情招商，充分发挥商会作用，积极促进威商回归。实施“威海英才计划”升级版，落实“人才+项目”科研资助计划，加大顶尖人才奖励资助和团队支持力度。实施“万名大学生聚集计划”，吸引高校毕业生来威就业创业。加快建设院士创新园、博士创新创业孵化基地等产业人才融合发展园区。实施“人才安居”工程，加强专家公寓、人才公寓建设。分产业领域举办“英创会”，搭建企业精准引才、合作引才、柔性引才平台。

　　加快对外贸易转型。积极培育贸易新业态新模式，扩大进出口贸易，推动出口市场多元化。深化国家服务贸易创新发展试点，打响“威海服务”的国际品牌。推动威海综合保税区创新发展。积极参与“数字丝绸之路”建设，打造“一带一路”国家云服务公共运营服务平台。推进外贸转型升级基地建设，培育出口产品质量安全区域性、行业性品牌。支持企业到境外建设生产基地、研发机构、营销网络及海外仓，扩大境外承包工程业务规模，推动省级以上境外经贸合作园区创建工作。

　　提升城市国际化环境。坚持世界眼光、国际标准、本土优势，制定城市国际化战略实施纲要和评价体系，实施城市功能国际化、城市经济国际化、城市人文国际化“三大策略”，推动开放城市向国际化城市迈进。提升学校和医院的国际化水平，增加城市各类外语标识，鼓励企业建设外文版网站，推动公共服务、社会管理与国际接轨。推进“三最”口岸建设。加强外事、侨务、台港澳工作，有效发挥国际友好城市作用，扩大各领域对外交流。加快国际经贸交流中心建设，增加国际展会，提升国际食品博览会、国际渔具博览会等展会层次，争取举办高层次的国际交流活动，提升城市国际影响力和知名度。

　　深化市场化改革。坚持向改革要动力，深化国资国企、财税金融、土地、市场准入、社会管理等领域改革。积极推进混合所有制改革，推动国有企业改革与重组。以市级投融资平台公司为重点，整合市属国有企业、国有资产、国有资源，组建城投、产业、文旅等大型企业集团，搭建国有资本投资运营平台，加快集团化、集约化发展。支持企业并购重组，加快推进8个战略合作项目。深化财税体制改革，调整完善市以下财政体制，健全更加灵活的转移支付机制。扩大预算绩效管理覆盖范围，对政府投资项目开展绩效前评估。坚持规范和鼓励并行，推进PPP项目运作，引导社会资本参与基础设施、公共服务等领域建设。建立“以亩产论英雄”的用地、用海考核机制，促进资源节约集约利用。

　　（四）聚焦区域协同创新，打造“千里海岸线，一条创新链”。落实创新驱动发展战略，以生态为基础，以创新为导向，以开放为引领，聚集各类创新资源，打造“千里海岸线，一条创新链”。

　　强化平台建设抓创新。推进创新平台化，构建开放、协同、高效的共性技术研发平台。加快建设国家区域创新中心，组建威海产业技术研究院，支持哈工大威海创新创业园建设，争创国家高性能医疗器械创新中心，依托工信部电子信息技术综合研究中心，加快建设海洋电子与智能装备研究院、网络安全及工业互联网安全创新中心等平台，构建“1+3+N”协同创新格局。建立工业设计中心梯次培育机制，新认定市级以上工业设计中心10家。

　　强化园区联动抓创新。充分发挥国家自主创新示范区的龙头引领作用，推动双岛湾科技城、东部滨海新城、南海新区、好运角旅游度假区等产业新城创新发展，加快建设十大高端特色产业园区，引导优质资源、创新项目加快聚集，打造空间联结、产业联动、要素聚集的创新链。实施总投资20亿元的园区配套提升工程，提高十大高端特色产业园区的项目承载能力，争取年内引进高端产业项目50个以上，建成投产20个以上。大力推进园区体制改革，开展开发区创新试点工作，促进开发区高质量发展。

　　强化企业培育抓创新。健全需求为导向、企业为主体的产学研一体化创新机制，实施小微企业治理结构和产业结构“双升”战略，推进科技型企业递进培育工程。实施科技企业孵化器专业聚焦行动计划，构建“众创空间—孵化器—加速器”企业孵化链条，提高大众创业万众创新水平。建设科技云平台，设立科技创新数据中心，促进科技资源联合共享。实施91项市级重点科技项目，提高科技成果转化能力。推进国家知识产权示范城市创建，力争万人有效发明专利拥有量由9.9件提高到11件。全社会研发投入占生产总值比重再提高0.1个百分点。

　　强化校企合作抓创新。支持高校院所来威海设立大学科技园、共建工业技术研究院，推动青岛大学威海创新研究院、乌克兰科技创新研究院威海分院加快建设。加强与驻威高校校地合作，订单式、精准化培养学生，为本地企业输送高素质、高技能实用型人才。完善产教融合、校企合作扶持政策，支持职业院校“靠高挂强”提升发展，引企入校开展混合所有制办学。适应产业发展需求，推进职业院校学科专业整合。探索设立职教园区、职教特色小镇，打造产教融合示范区。

　　（五）聚焦补齐短板，统筹加强基础设施建设。抢抓补短板、扩内需的有利机遇，突出“三个力保”，谋划推动一批事关长远的重大工程项目，进一步提升城市承载能力和发展后劲。

　　加强交通基础设施建设。围绕构建市域一体、国内通达和联通海外“三大交通圈”，推进19项重点交通项目。开工建设乳山口大桥、莱乳线乳山绕城段改建工程，加快推进文莱高速公路建设。环山路田和至温泉段改建工程主线通车，西线工程加快推进。新建改造农村公路500公里。启动莱荣高铁、荣成动车存车场建设，加快推动桃威铁路电气化改造。推进城市轨道交通项目建设前期工作。完成威海新机场规划选址和前期工作。

　　加强水利基础设施建设。按照“广蓄水、引客水、淡海水、用中水、治污水”的思路，实施10大类重点水利工程。完成龙角山水库增容工程、泊于水库蓄水工程，推进坤龙邢水库增容工程，开工建设黄垒河地下水库，开展母猪河地下水库前期工作，增强保水蓄水能力。规划论证南水北调南线工程，增强引水调水能力。结合全国节水型社会创新试点示范市建设，编制海水淡化专项规划，加强中水利用管网规划建设，实施荣成核电配套产业园海水淡化项目，建设龙山净水厂等17项城市给排水工程，增强供水节水能力。推进7条中小河流治理，实施崮山水库和89座小型水库除险加固工程，增强防汛防洪能力。

　　加快生态修复工程建设。坚持陆海统筹、水岸共治、管护并重，高标准实施生态系统保护和修复工程。实施海岸线修复三年行动计划，推进金海滩、爱莲湾等30项海岸带修复项目建设，实施“退建还海”工程，推动建设用海空间转化为海洋生态空间。实施山体修复三年行动计划，优先修复全域道路两侧破损山体，推进大岚山、里口山等80个矿山治理项目，治理面积260万平方米。推动“绿满威海·四季多彩”国土绿化行动，成片造林6万亩。实施河道治理三年行动计划，推进九龙河、钦村河等河道生态治理。加强湿地修复保护，加快五垒岛湾国家湿地公园建设。实施河库绿化三年行动计划，完成229条河流的河道绿化整治任务，让河更畅、水更清、岸更绿、景更美。

　　（六）聚焦城乡协调发展，打造乡村振兴齐鲁样板威海篇。坚持农业农村优先发展，实施乡村振兴战略三年行动计划，推动乡村产业振兴、人才振兴、文化振兴、生态振兴、组织振兴。

　　强化项目建设推动农业转型发展。坚持跨区域布局、跨行业整合、跨产业发展，突出“规划+”“特色+”“旅游+”“公路+”，三年实施40个重大工程项目，年内重点推进昆嵛田园综合体、临港区苹果小镇、伟德山现代休闲农业产业示范园区等示范项目，培育壮大农业“新六产”。大力发展精致农业，坚持藏粮于地、藏粮于技，实施耕地质量提升计划，切实抓好粮食生产；推动特色农产品品种优化、品质提升、加工升级，打造4个万亩、4个五千亩现代农业产业园，争创国家级特色农产品优势区。大力发展绿色生态农业，建设生态循环农业示范基地，推广水肥一体化技术7万亩、测土配方施肥技术300万亩，推动化肥农药用量和强度“双减”，提升农产品质量安全水平，着力增加优质绿色农产品供给。大力发展品牌农业，新发展农业标准化基地4万亩，新培育知名农产品区域公用品牌2个、企业产品品牌10个，打响“威海农产·品味自然”“威海苹果·甜蜜诱惑”品牌。

　　发展美丽经济带动美丽乡村建设。按照“生态美、环境优，生产美、产业强，生活美、家园好”的思路，将景区理念与旅游元素融入美丽乡村建设，突出古村古韵、渔村渔家、山海情怀、特色农业等文化元素，创建省级示范村20个，培育市级示范村69个，努力把示范村建成特色景点，把农民庭院建成精致民宿，把交通沿线建成风景长廊，集中连片打造美丽乡村示范带。实施乡村传统工艺振兴计划和乡村记忆保护工程，保护传统民居、古迹村落和传统乡村。实施好美丽村居建设“四一三”行动，新增4个省级示范村。高标准推进农村“七改”工程，扎实开展农村饮水安全攻坚行动，改善529个村供水条件；建立农村改厕后续管护长效机制，支持荣成市开展农村生活垃圾分类试点；加强农村散煤燃烧治理，新增农村清洁供暖用户5000户。深入开展农村人居环境整治行动，提升农村环境绿化、净化、美化水平。

　　深化改革激发农业农村活力。探索农村土地“三权分置”有效形式，推进农村闲散土地盘活利用，引导和推动土地规模流转。开展农村集体资产股权质押试点，推进954个村集体经济组织产权制度改革。大力培育家庭农场、农民合作社等新型经营主体，建设农业社会化综合服务中心15处，探索农业新型集体化经营模式。实施乡村振兴产业人才支持计划，建立新型职业农民培训基地，培育现代职业农民500人以上。推进乳山市全国农村综合性改革试点试验、文登区农业社会化服务体系建设探索试验、汪疃镇乡村振兴综合试点工作。健全涉农资金统筹整合长效机制，设立乡村振兴专项资金池。创新涉农金融产品服务，引导更多金融和社会资本投向乡村振兴。推进文明乡村、法治乡村、平安乡村建设，打造充满活力、和谐有序的善治乡村。

　　（七）聚焦打好三大攻坚战，守住发展底线红线。坚持抓重点补短板强弱项，针对突出问题，打好重点战役。

　　以底线思维打好防范化解重大风险攻坚战。健全金融风险防范、监测预警和应对处置机制，积极稳妥化解不良贷款和债券兑付风险，严厉打击逃废银行债务行为和各类非法金融活动。规范政府举债融资机制，有序处置存量政府债务。深化安全生产双重预防体系建设，加强消防、森林防火、涉氨企业、道路交通、海上安全、校园安全、食品药品安全等重点领域专项治理。深入开展化工产业安全生产转型升级专项行动。加强自然灾害防治体系建设，提高应急管理水平和防灾减灾救灾能力。推进平安威海建设，深化扫黑除恶专项斗争，加强禁毒、反恐、网络安全工作。健全“1+7”社会治理体系，加强诉调对接工作，完善矛盾纠纷多元化解机制，打造四位一体的立体化社会治安防控体系，维护社会和谐稳定。进一步做好民族工作，构建积极健康的宗教关系。

　　以绣花功夫打好精准脱贫攻坚战。实施打赢脱贫攻坚战三年行动，强化贫困人口动态监测、动态调整，研究解决那些收入水平略高于建档立卡贫困户的群体缺乏政策支持等新问题。加强“老病残”等困难群体、“插花式”贫困人口帮扶，减少和防止贫困人口返贫。整合提升扶贫产业项目，扩大“富民生产贷”等金融扶贫规模。开展扶贫扶志行动，加强技能培训，增强脱贫内生动力。加强对口支援和东西扶贫协作。

　　以铁骨担当打好污染防治攻坚战。高质量推进中央环保督察及“回头看”、国家海洋督察、省级环保督察反馈问题和群众举报问题整改，坚决杜绝表面整改、虚假整改、敷衍整改。按照“1+1+8”工作部署，大力推动“四减四增”。打好蓝天保卫战，建成覆盖镇街的环境空气质量监测网络，开展污染源扩散预测和成因解析，实施臭氧、移动污染源、扬尘污染专项整治，确保环境空气质量继续稳定达到国家二级标准。打好碧水保卫战，严格落实“三长制”，开展12处集中式饮用水水源地规范化建设，实施黄垒河达标治理，加强河流综合整治，规范入海排污口设置。打好净土保卫战，强化危险废物规范管理，建成工业固体废物处置中心。加强农业面源污染治理，推进农业废弃物无害化处理和资源化利用。打击“洋垃圾”入境。开展生态设计企业试点工作。发展装配式建筑和绿色建筑。编制好生态保护红线、环境质量底线、资源利用上线和环境准入负面清单“三线一单”，推进生态红线勘界定标。加强生态文明宣传教育，推进网格化环境监管体系建设，筑牢环境保护基层基础。

　　（八）聚焦保障改善民生，全面提高公共服务水平。紧紧围绕解决民生领域的不平衡不充分问题，突出保基本兜底线，从群众关心的事情抓起，实实在在为群众解难题、增福祉。

　　优先发展教育事业。推动学前教育优质普惠发展，新建改扩建普惠性幼儿园20所，增加学位5010个，普惠性幼儿园覆盖率提高2个百分点。推动义务教育优质均衡发展，新建改扩建中小学校15所，增加学位1.09万个，着力解决中小学生课外负担重等问题。推动普通高中优质特色发展，深化新高考背景下课程教学改革，加快课程基地和特色高中建设。推动特殊教育优质融合发展，启动市特殊教育学校迁建工程。推动民办教育优质规范发展，推进民办学校分类管理改革，培育放心消费示范培训机构。

　　提高社会保障水平。把稳就业摆在突出位置，实施就业优先政策，重点解决好高校毕业生、农民工等群体就业。改革就业培训机制，年内实施就业创业培训2万人次，新增城镇就业3.3万人。提高居民基础养老金标准，建立居民长期护理保险制度，深化医保支付方式改革。推行政府购买社会救助服务，进一步提高城乡低保标准和特困人员基本生活标准。坚持房子是用来住的、不是用来炒的定位，构建房地产市场健康发展长效机制，培育发展住房租赁市场，完善住房市场体系和住房保障体系。

　　加快建设健康城市。深化医药卫生体制改革，完善现代医院管理制度，深入推进分级诊疗，加快建设医联体、医共体。坚持做强龙头、做优专科，探索建设医疗集团、医学中心，鼓励社会力量建设高端特色医院。加快建设全民健康信息平台，提升家庭医生签约服务质量。建设全市出生缺陷防控中心、危重儿童救治中心、危重孕产妇救治中心，强化重大疾病预防控制。完善养老护理体系，探索虚拟养老院模式，依托12349服务平台为困难老年人购买居家养老服务。推进镇村敬老院社会化改革，建设12处镇街养老服务中心。实施15个医养结合项目，新增医养结合养老床位1200张。广泛开展全民健身活动，做精做活铁人三项世界杯赛、亚洲霍比帆船锦标赛、全国海钓锦标赛等品牌赛事。

　　丰富群众精神文化生活。深化全国公共文化服务体系示范区创建，实施“智慧文化”项目，升级文化设施和服务。新建城市书房6处，擦亮“书香威海”全民阅读品牌。举办第三届威海文化惠民消费季，打造威海特色文化消费品牌。培育新型文化业态，打造“中国油画小镇”等文化品牌。挖掘整理档案、史志资源，加强历史文化传承保护和合理利用，打造100个历史文化展室示范点和100个儒学讲堂示范点。抓好胶东（威海）党性教育基地深化提升工作，传承和弘扬红色基因。新建社会组织服务中心24处、社区社会组织服务室72个。深化文明市民培育工程，开展“铸魂”“养德”“修身”三项活动。推进新时代文明实践中心试点建设，广泛开展移风易俗、弘扬时代新风行动。深化“君子之风·美德威海”建设，开展“美德威海·友善社区”创建，打造“志愿威海”公益品牌，推动社会主义核心价值观落细落小落实。

　　巩固军政军民团结。贯彻军民融合深度发展战略，打造军民融合协同创新中心和技术成果转移中心，培育军地通用装备制造、军民两用新材料等军民融合优势产业，争创国家军民融合创新示范区。扎实开展全国双拥模范城创建工作，加强国防动员、民兵预备役、人民防空等工作。严格落实各项政策，做好军转干部、退役士兵安置和权益保障工作。

　　各位代表！人民对美好生活的向往，就是我们的奋斗目标。在广泛征求群众意见建议的基础上，今年确定办理12类民生实事，以此为切入点，着力解决群众关心的痛点难点堵点问题，实实在在增强群众的获得感、幸福感。

三、建设人民满意的服务型政府

　　始终把以人民为中心作为根本价值取向，树牢“四个意识”，坚定“四个自信”，做到“两个维护”，站稳政治立场，强化责任担当，把今年作为“工作落实攻坚年”，坚持高点定位，主动寻标对标，以雷厉风行、专业务实的作风狠抓工作落实，以优质高效的服务营造一流营商环境，调动全市干部群众的积极性和主动性，激发广大企业的活力和创造力，推动各项工作取得新突破。

　　（一）持续优化服务，提高企业和群众办事的便捷度和满意度。坚持“换位思考、主动服务、有求必应、无需不扰、结果评价”，从看得见的前台和看不见的后台两个维度，持续深化“一次办好”改革，营造精简高效政务生态。推进“数字政府”建设，深化“互联网+政务服务”，继续推动政务信息资源整合共享，进一步打通部门间数据壁垒，用共享数据代替办事材料。推进“智慧民生”自助服务终端“一端多用”，让群众少跑腿、好办事。健全12345政务服务热线工作机制，完善考核评价体系，提高为民服务水平。开展政务服务事项全链条优化联办，实行“前台综合受理、后台分类审批、统一窗口出件”，实现“一站式”办结。深入推进咨询导办、贴心帮办、全程代办服务，提升政务服务规范化、标准化、便利化水平。落实市级领导联系企业和项目制度，开展“千名干部联系千家企业”行动，搭建政府与企业直通车，为企业提供“店小二”“保姆式”最优质服务。

　　（二）持续规范监管，营造更加稳定公平透明、更可预期的市场环境。切实转变政府职能，强化事中事后监管，凡是市场能自主调节的就让市场来调节，凡是企业能干的就让企业干。强化“双随机、一公开”监管，严格规范行政执法行为，推动监管全流程信息化、智能化。落实市场准入负面清单制度，打破行业垄断和市场壁垒，对各类市场主体一视同仁、公平对待，在政策执行方式上不搞简单化、“一刀切”。开展企业“法律体检”活动，为企业提供优质法律服务。深入推进信用监管，完善行业信用“红黑名单”和部门联合奖惩机制，强化信用积分评价结果运用，建立“事前信用承诺、事中分类监管、事后联合奖惩”的新型市场监管模式，让信用威海成为最好的营商环境。

　　（三）持续创优环境，构建亲清新型政商关系。开展企业管理人员轮训，为企业送思想、送政策、送信心，提高政策的知晓度、精准度和实效性。大力弘扬企业家精神和工匠精神，激发企业创新活力和创造潜能。开展民营企业“青蓝接力”行动，助力年轻一代企业家成长。健全企业家参与涉企政策制定机制和政策执行效果第三方评估机制，避免出现“桃子政策”“抽屉政策”，实实在在增加企业获得感。及时回应和解决企业诉求，构建平等保护各类市场主体产权的长效机制，在全社会营造尊重企业家、保护企业家、支持企业发展的浓厚氛围。

　　（四）持续健全机制，主动接受各方监督、规范行政权力运行。坚持依法行政，加强政府立法工作，扎实开展市县法治政府示范创建，将政府活动全部纳入法治轨道、置于阳光之下。自觉接受市人大及其常委会法律监督、工作监督，健全依法决策机制，重大事项主动提请审议，重大决策出台前及时报告，落实好预算执行、审计查出问题整改、国有资产管理、重要规划编制等报告制度。自觉接受政协民主监督，健全与人大代表、政协委员的沟通联络机制，广泛听取民主党派、工商联、无党派人士和人民团体意见，高质量办理人大代表建议和政协提案。主动接受社会监督和舆论监督，全面推进政务公开，畅通政企、政民沟通渠道，及时回应社会关切。

　　（五）持续转变作风，营造风清气正、廉洁高效的政务环境。严格落实全面从严治党责任，严守意识形态主阵地。适应政府机构改革要求，加强干部教育培训，提高履职能力。落实激励机制和容错纠错机制，激励干部担当作为，鼓励创造性贯彻落实。推进审计监督全覆盖，加强对重点领域、关键环节、重要岗位权力运行的监督制约。持之以恒正风肃纪，坚决纠正“四风”，力戒形式主义、官僚主义，巩固拓展落实中央八项规定精神成果。深入推进政府系统廉政建设和反腐败斗争，严肃查处侵害群众利益的行为和腐败问题，以为民务实清廉的作风推动各项工作落实。

　　各位代表！带着总书记的嘱托和期望，我们踏上新的征程。使命在心、重任在肩，唯有实干才能不负担当。让我们紧密团结在以习近平同志为核心的党中央周围，在市委的坚强领导下，不忘初心、牢记使命，以更加饱满的激情、更加务实的作风、更加勤勉的工作，不断开创各项工作新局面，努力争当全省走在前列排头兵，为加快建设“精致城市·幸福威海”而努力奋斗！
